# Bioinformatics Analysis Combined With Experiments Predicts PUDP as a Potential Prognostic Biomarker for Hepatocellular Carcinoma Through Its Interaction With Tumor Microenvironment

**DOI:** 10.3389/fonc.2022.830174

**Published:** 2022-03-08

**Authors:** Jiahao Yu, Weirui Zhang, Dawei Ding, Yinan Hu, Guanya Guo, Jingbo Wang, Ying Han

**Affiliations:** ^1^ State Key Laboratory of Cancer Biology, Xijing Hospital of Digestive Diseases, The Fourth Military Medical University, Xi’an, China; ^2^ Department of Biomedical Engineering, The Fourth Military Medical University, Xi’an, China

**Keywords:** HCC, PUDP, bioinformatics, prognosis, tumor microenvironment

## Abstract

Hepatocellular carcinoma (HCC) is one of the deadliest tumors in the world and is notorious for poor prognosis. There is mounting evidence that pseudouridine performs key functions in the initiation and progression of several cancers. A previous study demonstrated that Pseudouridine 5’-phosphatase (PUDP) may be a novel prognostic biomarker in colorectal cancer. However, in the past, we have paid little attention to PUDP and we are still not clear about its function and role in cancer. In this study, a pan-cancer analysis of PUDP expression and prognosis was performed firstly using The Cancer Genome Atlas (TCGA) and Genotype-Tissue Expression (GTEx) data and we found that PUDP may be a potential oncogene for HCC. Then the most potential upstream microRNA contributing to PUDP was identified as let-7c-5p through expression analysis, correlation analysis, and survival analysis. Subsequently, the result of single cell RNA sequencing (scRNA-seq) demonstrated that PUDP was significantly highly expressed on malignant cells. In addition, there are significantly positive correlations between PUDP and tumor immune cell infiltration, biomarkers of immune cells, and immune checkpoint expression, especially with tumor-promoting immune cells such as T cell regulatory (Treg), Myeloid-derived suppressor cell (MDSC), cancer-associated fibroblast (CAF). Moreover, we found the methylation level of three loci was positively correlated with PUDP expression and four loci were negatively correlated. 15 pairs of HCC and normal adjacent tissues from HCC patients who were treated at our center were used to verify the results of the bioinformatics analysis and the results of experiments are similar to the bioinformatics analysis. Our study demonstrated that HCC patients with high PUDP expression are less likely to benefit from immunotherapy, and in addition, we explored the relationship between PUDP and anticancer drugs. Finally, we explored the clinical relevance of PUDP, identified PUDP as an independent risk factor for HCC patients and constructed a prognostic model, used International Cancer Genome Consortium (ICGC) data to do external validation. Collectively, our study demonstrated that high expression of PUDP suggested a poor prognosis and low response to immunotherapy, providing new insight into the treatment and prognosis of HCC.

## Introduction

Hepatocellular carcinoma(HCC) is a highly aggressive and inflammation-associated cancer, accounts for 70%-85% of the total liver cancer burden (1). The incidence and the mortality of HCC respectively ranks 6th, 3rd worldwide and the new cases of HCC were estimated that up to 906,000 and 830,000 related deaths occur each year (2). The initiation and progression of HCC depend on lots of risk factors, such as alcohol abuse, hepatitis B virus infection, obesity, and immune microenvironment (2–5). In recent years, significant advances have been made in the diagnosis, treatment, and prognosis of HCC, such as the PD-L1 inhibitor atezolizumab, combined with the anti-angiogenic agent bevacizumab, which has achieved success in the first-line treatment of advanced HCC, however, treatment outcomes for HCC patients remain unsatisfactory (6). Therefore, it is necessary for us to find new and effective prognostic biomarkers.

Pseudouridine 5’-phosphatase (PUDP), also called HDHD1 or HDHD1A, encodes a member of the haloacid dehalogenase-like (HAD) hydrolase superfamily, was a phosphatase specifically involved in dephosphorylation of pseudouridine 5’-phosphate (7). Pseudouridylation, the most common type of RNA epigenetic modification in biosome, and was discovered to play a critical role in the translation control of stem cells with implications for development and disease (8). According to the latest findings, Pseudouridylation alters splicing, while genes that control essential processes, such as metabolism, cell proliferation and apoptosis, are globally altered in HCC through alternative splicing (9, 10). Furthermore, pseudouridine was reported may be a potential biomarker for kidney disease, which is characterized by a decline in kidney function and the occurrence of chronic kidney disease (11). Some pseudouridine metabolism-related enzymes, such as dyskerin pseudouridine synthase 1(DKC1), have been reported that correlate with the prognosis of HCC patients (12). Past researches revealed that the expression level of PUDP may be a prognostic biomarker for colorectal cancer and demonstrated that the overexpression of PUDP conferred 2-deoxyglucose(2DG) resistance in HeLa cells, suggesting that it may be a conserved regulator of 2DG resistance and could interfere 2DG-based chemotherapies (13, 14). However, the biological function of PUDP remains poorly understood and a thorough study on the expression, prognosis, and mechanism of PUDP in HCC is absent.

In this study, the expression level of PUDP in pan-cancer was analyzed firstly. According to the results of survival analysis and expression analysis, PUDP was identified to be linked with the prognosis of HCC patients. Then, we incorporated 711 HCC samples and 475 normal tissues from The Cancer Genome Atlas (TCGA) database, GSE39791 and GSE25097 to analyze the differential expression of PUDP in HCC patients. And we verified the differential expression of PUDP by using qPCR, it was also validated in the level of protein using the HPA database. Next, the regulation of microRNAs(miRNAs) with PUDP was explored in HCC. We also did the mutation and correlation analysis between PUDP and common immune checkpoint in HCC. In addition, we analyze the response of immunotherapy for high PUDP expression patients and low PUDP expression patients. Subsequently, we demonstrated the correlation between PUDP expression and immune infiltration, clinical staging, clinical prognosis, and sensitivity to anticancer drugs. Finally, the prognostic model was constructed using data from the TCGA database and was validated using data from the International Cancer Genome Consortium (ICGC). In summary, our findings suggest that PUDP is an independent risk factor and may be a novel prognostic biomarker for HCC patients.

## Materials and Methods

### Ethics Statement

Tumor samples were collected from the tumor tissue bank of Xijing Hospital. The samples were stored in liquid nitrogen before use and were collected in accordance with the ethical standards of the 1964 Declaration of Helsinki. This study was approved by The Xijing Hospital’s Ethics Committee.

### Data Download, Process, and Analysis

The mRNA expression data of 33 cancer types were downloaded from TCGA official website (https://portal.gdc.cancer.gov/) and then were used to perform the differential expression analysis for PUDP in 33 cancer types by using R package “limma” (15). HCC RNA sequencing data (GSE25097 and GSE39791) came from Gene Expression Omnibus(GEO) database and were used to analyze the differential expression of PUDP in HCC patients after normalization. GSE25097 includes the data of 268 HCC tumor tissues and 243 normal tissues. GSE39791 includes the 72 HCC tumor tissues and paired adjacent non-tumor tissues. The baseline information of HCC patients in the TCGA database is shown in **Table 1**.

**Table 1:** Baseline information of HCC patients from the TCGA database.

Characteristic	levels	Overall
n		374
T stage, n (%)	T1	183 (49.3%)
	T2	95 (25.6%)
	T3	80 (21.6%)
	T4	13 (3.5%)
N stage, n (%)	N0	254 (98.4%)
	N1	4 (1.6%)
M stage, n (%)	M0	268 (98.5%)
	M1	4 (1.5%)
Pathologic stage, n (%)	Stage I	173 (49.4%)
	Stage II	87 (24.9%)
	Stage III	85 (24.3%)
	Stage IV	5 (1.4%)
Tumor status, n (%)	Tumor free	202 (56.9%)
	With tumor	153 (43.1%)
Gender, n (%)	Female	121 (32.4%)
	Male	253 (67.6%)
Age, n (%)	<=60	177 (47.5%)
	>60	196 (52.5%)
Histologic grade, n (%)	G1	55 (14.9%)
	G2	178 (48.2%)
	G3	124 (33.6%)
	G4	12 (3.3%)
AFP(ng/ml), n (%)	<=400	215 (76.8%)
	>400	65 (23.2%)
OS event, n (%)	Alive	244 (65.2%)
	Dead	130 (34.8%)
Age, median (IQR)		61 (52, 69)

### Differential Expression and Survival Analysis

Besides the differential expression analysis for PUDP in 33 cancer types using the data from the TCGA database, we also analyzed the expression level of PUDP in pan-cancer by using TIMER2.0 (http://timer.cistrome.org/), ONCOMINE (https://www.oncomine.org/resource/main.html) and GEPIA (http://gepia.cancer-pku.cn/). Kaplan-Meier(K-M) survival analysis was performed by using the R package “survminer” (https://cran.r-project.org/web/packages/survminer/index.html) and R package “survival” (https://cran.r-project.org/web/packages/survival/index.html). We then summarized the results of the K-M analysis and did separate K-M analyses for HCC and Pancreatic adenocarcinoma (PAAD).

### The Protein Expression Level for PUDP in HCC Patients

The Human Protein Atlas (HPA, https://www.proteinatlas.org/), a Swedish-based program, aims to figure all the human proteins. Data from the HPA database is freely available for all researchers and we downloaded the immunohistochemistry images of PUDP expression from the HPA database.

### qPCR

In accordance with the manufacturer’s protocol, we extracted total RNA from HCC samples with the TRIzol reagent (Ambion). β-Actin was used as an internal control to normalize mRNA expression. The primer sequences for PUDP were GCAATGACAACTGATAAAGCGAGATG (Forward primer 5’-3’), GCTAACAAGGAGTGCTCATCAAAAAC (Reverse primer 5’-3’) and the primer sequences for β-actin were CTCCATCCTGGCCTCGCTGT (Forward primer 5’-3’), GCTGTCACCTTCACCGTTCC (Reverse primer 5’-3’). The pathological stage for most HCC samples were stage II and stage III.

### Identification of Differentially Expressed Genes

According to the expression of PUDP in HCC patients, high and low PUDP expression groups were divided based on median value. Then we analyzed the significantly differentially expressed genes(DEGs) between the high and low expression groups by using the R package “DESeq2” (16). The results were displayed in the form of a volcano map and heat map. The threshold value for significant DEGs was adjust *P-value*<0.05 and *|Log fold change(FC) |*≥2.0.

### Enrichment Analysis of PUDP

We used the “clusterprofiler” package to do Gene Ontology(GO) enrichment and Kyoto Encyclopedia of Genes and Genomes(KEGG) analysis (17). The threshold values were identified with corrected *P*<0.01 and a false discovery rate(FDR) <0.05. Gene set enrichment analysis (GSEA) was performed using the gene set “c2.cp.v7.2.symbols.gmt” which were downloaded from the Molecular Signatures Database (MSigDB, http://software.broadinstitute.org/gsea/msigdb/index.jsp).

### ScRNA-Seq Analysis

Single-cell RNA sequencing (scRNA-seq) demonstrated the distribution and expression of PUDP in HCC patients. Data of scRNA sequencing were obtained from GSE125449 and Tumor Immune Single-cell Hub (TISCH) was used to perform analysis (18).

### Mutation and Correlation Analysis of PUDP With Common Immune Checkpoints

We analyzed the mutation status and correlation of PUDP with common immune checkpoints in HCC using cBioPortal for Cancer Genomics (http://www.cbioportal.org) (19, 20). The results of the correlation analysis between PUDP and four common immune checkpoints of HCC are presented in the form of scatter plots.

### Correlation of PUDP With the Infiltration of Immune Cells

We used the Timer2.0 website to analyze the infiltration relationship between PUDP and 6 types of immune cells (21). Besides, we explored the immune infiltration distribution between different somatic copy number variations(sCNA) status of the PUDP with T cell regulatory.

### Prediction of the Immunotherapeutic Response

Tumor Immune Dysfunction and Exclusion (TIDE) algorithm was used to predict the potential immunotherapeutic response using TCGA RNA sequencing data and the corresponding clinical information of HCC patients (22).

### Candidate MiRNA Prediction

StarBase (http://starbase.sysu.edu.cn/), an open-source database, identifies millions of miRNA-ncRNA, miRNA-mRNA, RBP-RNA, and RNA-RNA interactions from multi-dimensional sequencing data (23). StarBase predicts miRNA-mRNA interactions by multiple prediction tools such as PITA, RNA22, miRmap, DIANA-microT, miRanda, PicTar and TargetScan. Those that meet the screening of more than two prediction tools are considered as potential upstream regulatory miRNAs of PUDP.

### DNA Methylation Analysis

We estimated and visualized DNA methylation of PUDP in HCC by using the MEXPRESS web server (https://mexpress.be) (24, 25). MEXPRESS focuses on correlation analysis and visualization using information such as gene expression profiles, DNA methylation sites, and clinical data from the TCGA database. The predesignated methylation probes for each gene were taken into consideration.

### Correlations of the Expression Levels of PUDP With Anticancer Drug Sensitivity and Clinical Stage

The CellMiner database (https://discover.nci.nih.gov/cellminer/home.do) which is an open database, was performed to analyze the relationship between 23,101 chemical compounds, including 218 drugs approved by FDA and 574 drugs tested by clinical trials, and the expression level of PUDP (26). R package limma and R package impute (http://www.bioconductor.org/packages/release/bioc/html/impute.html) were used in the analysis (27).

### Establishment and Validation of the Clinical Prognostic Model

The clinical information of 337 HCC patients such as sex, age, clinical stage, and the expression level of PUDP were downloaded from the UCSC Xena website (http://xena.ucsc.edu/) to establish the prognostic model. The LIRI-JP cohort data which were downloaded from the ICGC database (https://dcc.icgc.org/) were used to perform external validation.

Risk score of HCC patients was performed by Cox regression analysis and R package “rms”, R package “survival” were used to construct the nomogram. Harrell’s concordance index(C-index) and calibration curve were used to do internal validation of the prognostic model.

### Statistical Analysis

For K-M survival analysis, the log-rank test was utilized. The receiver operating characteristic (ROC) curve and the area under the curve (AUC) were conducted using R package “survival ROC” and “pROC” (28, 29). In calculating the relationship between immune cells, co-expressed genes, and the expression level of PUDP, we used the Spearman statistical method. All statistical analyses were performed by R software, version 4.1.0.

## Results

### PUDP Expression Profiles in Pan-Cancer

**Figure 1:**
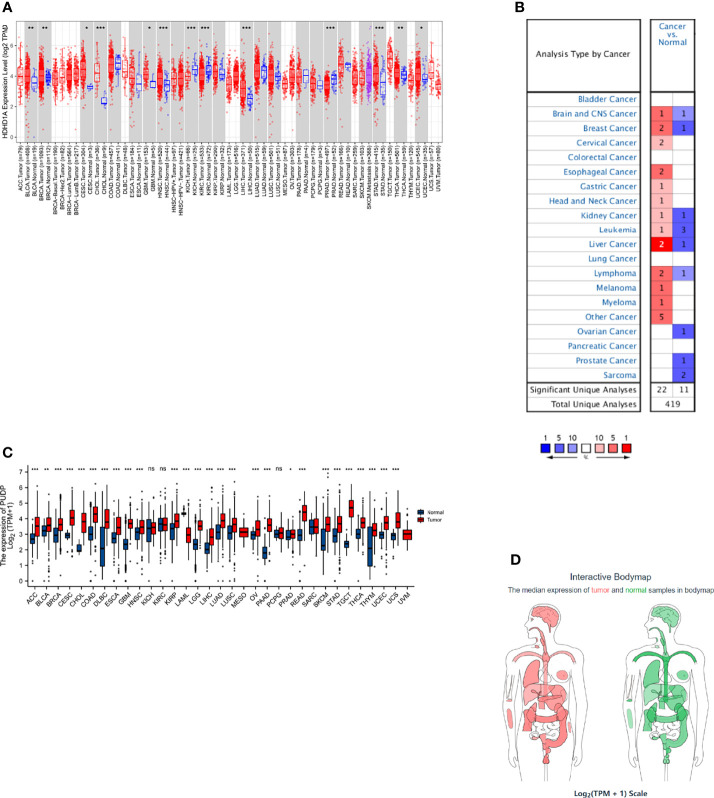
The differential expression of PUDP in Pan-Cancer. We used Timer2.0, Oncomine database to identify the differential expression of PUDP in Pan-Cancer, and performed differential expression analysis using data from TCGA and GTEx, and finally used the GEPIA database to show an interactive bodymap of PUDP expression in humans. **(A)** Using Timer2.0 database to identify the differential expression of PUDP; **(B)** Using Oncomine database to identify the differential expression of PUDP; **(C)** Using data from TCGA and GTEx to identify the differential expression analysis of PUDP; **(D)** An interactive bodymap of PUDP in human using GEPIA database. *P < 0.05, **P < 0.01, ***P < 0.001; ns, no significant difference in statistics.

We first analyzed the differential expression of PUDP between tumor and adjacent non-tumor tissues in pan-cancer and used different data sources and databases to ensure the reliability of the results that we analyzed. In a pan-cancer analysis using the Timer2.0 database, we found that PUDP was significantly up-regulated expressed in 11 types of tumors (**Figure 1A**). We then used the Oncomine database to perform differential expression analysis of PUDP in tumor and normal tissues (**Figure 1B**). In addition to using the database analysis, we downloaded RNAseq data from TCGA and GTEx and transformed them into the form of log2, and the results are shown in **Figure 1C**. We finally used the GEPIA database to do an interactive bodymap on the expression of PUDP in various human organs. Red represents expression in tumor tissues, green represents expression in normal tissues, and the shade of color represents the degree of expression (**Figure 1D**). The results of the analysis showed that PUDP is differentially expressed in most tumors and adjacent non-tumor tissues, but whether they all have a prognostic value according to the expression level of PUDP remains unknown.

### Relationship Between PUDP Expression and Prognosis in Pan-Caner

**Figure 2:**
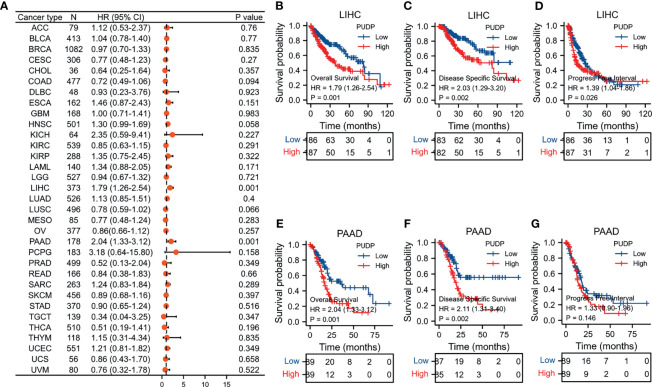
Survival analysis in Pan-Cancer and exploring the prognostic value in PAAD and HCC of PUDP. **(A)** The prognostic value of PUDP in Pan-Cancer; **(B–D)** Prognosis analysis of PUDP in HCC; **(E–G)** Prognosis analysis of PUDP in PAAD.

Using gene expression and clinical data from the TCGA, survival analysis was performed to analyze the relationship between PUDP expression and prognosis. We summarized the results of analyses and found that PUDP had a significant prognostic value in PAAD and HCC, with a p-value less than 0.001 in both (**Figure 2A**). Employing OS, DSS, and PFI as outcome endpoints, we determined the predictive value of PUDP in HCC and PAAD (**Figures 2B–G**). The results demonstrated that PUDP had a significant prognostic value in HCC regardless of which of OS, DSS, PFI was used as the outcome endpoint, while hazard ratio (HR) value greater than 1 suggested that it was a risk factor. Our findings demonstrated that high PUDP expression correlates a poor prognosis of HCC patients.

### The Analysis of Differential Expression of PUDP in HCC

**Figure 3:**
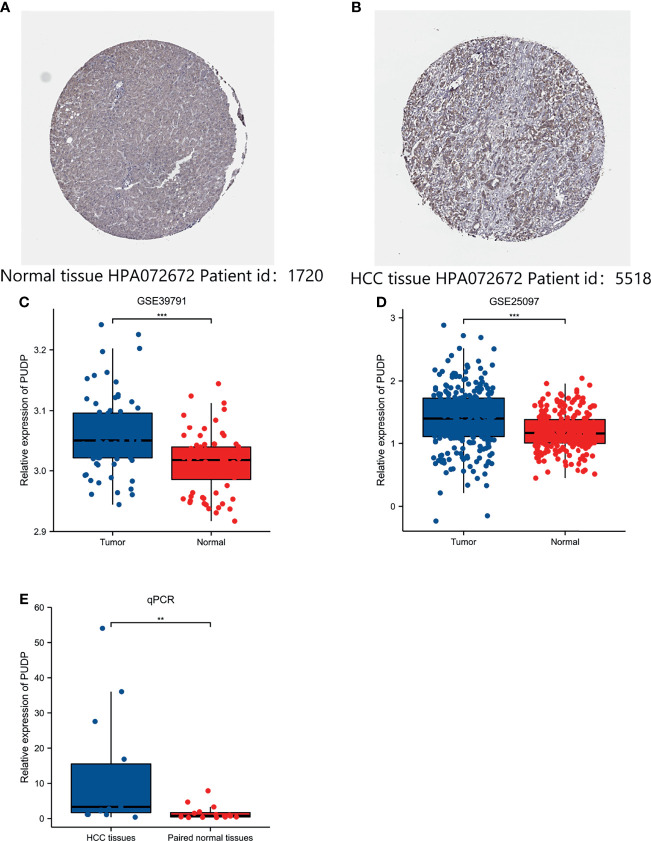
Exploring the expression of PUDP in HCC. **(A)** The expression of PUDP in normal liver tissues on the level of protein; **(B)** The expression of PUDP in HCC tissues on the level of protein; **(C)** Expression analysis of PUDP using GSE39791; **(D)** Expression analysis of PUDP using GSE25097; **(E)** The result of qPCR. *P < 0.05, **P < 0.01, ***P < 0.001.

In our previous analysis, we found that PUDP may be a novel prognostic biomarker for HCC. Then, we used two datasets, GSE39791 and GSE25097, as well as the HPA database, to re-identify the differences of PUDP expression between HCC tissues with adjacent non-tumor tissues at the mRNA and protein levels. The results demonstrated that PUDP has a significant differential expression on the level of mRNA and protein between HCC tissues and adjacent non-tumor tissues (**Figures 3A–D**). Finally, we collected tumor and paired adjacent non-tumor tissues from 15 HCC patients from the tumor tissue bank of Xijing Hospital for qPCR assay, and again verified this differential expression on mRNA level (**Figure 3E**). All the above analyses suggested that there was a significant difference in the expression of PUDP in HCC tissues and adjacent non-tumor tissues.

### Identification of DEGs Associated With PUDP Expression in HCC

**Figure 4:**
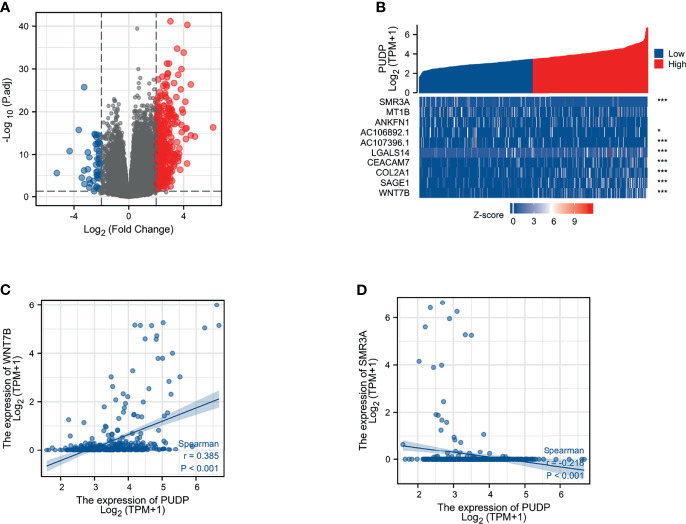
Identification of the DEGs between PUDP high and low expression groups in HCC. **(A)** Volcano map of DEGs that 453 genes were upregulated and 48 genes were downregulated; **(B)** Correlation heat map of the top 5 up- and down-regulated genes with PUDP; **(C)** Correlation of PUDP and Wnt7B in patients with HCC; **(D)** Correlation of PUDP and SMR3A in patients with HCC. (*p < 0.05, ***p < 0.001).

According to the expression profile of PUDP, we separated HCC patients into two groups: high PUDP expression and low PUDP expression, with the low PUDP expression group serving as the reference group for DEGs analysis. We found that 453 genes were upregulated and 48 genes were downregulated with a strict cut-off of *p*<0.05 and *|Log fold change(FC) |*≥2.0 (**Figure 4A**). We took the top 5 up-regulated and down-regulated genes according to the size of LogFC values and plotted the correlation heat map (**Figure 4B**). We then analyzed the correlation of PUDP with Wnt7B and SMR3A in patients with HCC (**Figures 4C, D**). The results suggested that PUDP had a strong positive correlation with Wnt7B, while a strong negative correlation with SMR3A.

### Enrichment Analysis of PUDP in HCC

**Figure 5:**
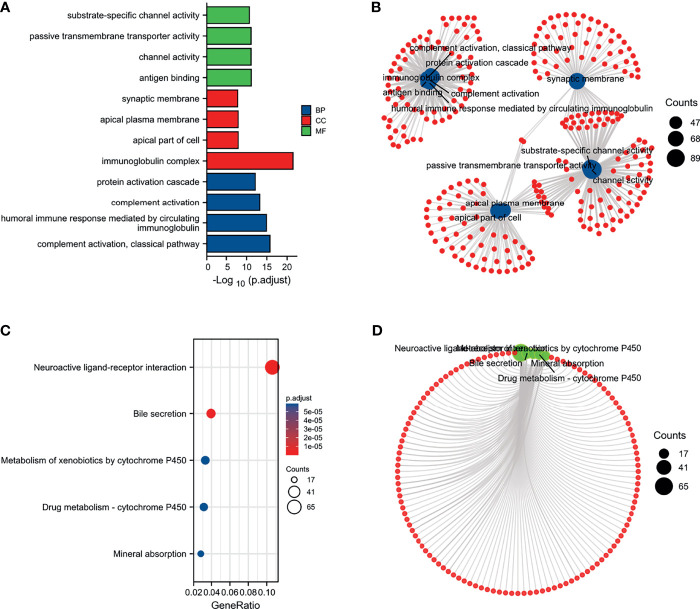
Enrichment analysis of PUDP. **(A, B)** The results of GO analysis; **(C, D)** The results of KEGG analysis.

We performed a functional enrichment analysis of the PUDP. The results of the molecular function of GO analysis demonstrated that PUDP was closely associated with substrate-specific channel activity, channel activity, passive transmembrane transporter activity, and antigen binding. And GO analysis showed that the PUDP was closely associated with biological processes such as protein activation cascade, complement activation, humoral immune response mediated by circulating immunoglobulin and complement activation, classical pathway (**Figures 5A** **, B**). The results of KEGG functional analysis suggested that PUDP mainly affects the neuroactive ligand-receptor interaction, bile secretion (**Figures 5C, D**).

Furthermore, the GSEA of DEGs revealed that the enrichment of DEGs were correlated with the NABA MATRISOME, REACTOME CELL CYCLE CHECKPOINTS, REACTOME CELL CYCLE MITOTIC, REACTOME CELL CYCLE, AND REACTOME M PHASE (**Supplementary Figures 1A, B**).

### ScRNA Analysis of PUDP Expression on Different Cells of HCC

We utilized the HCC scRNA sequencing results from the GSE125449 dataset to investigate the expression of PUDP in different cells of HCC. Our work indicated that in HCC, PUDP was expressed in exhausted CD8 T cells (CD8Tex), monocytes or macrophages, endothelial cells, fibroblasts, malignant cells, hepatic progenitor cells, and especially in malignant cells with significantly high expression (**Supplementary Figure 2**). The findings suggested that PUDP expression is related to the tumor microenvironment in HCC.

### Immune Infiltration and Drug Sensitivity Analysis of PUDP

**Figure 6:**
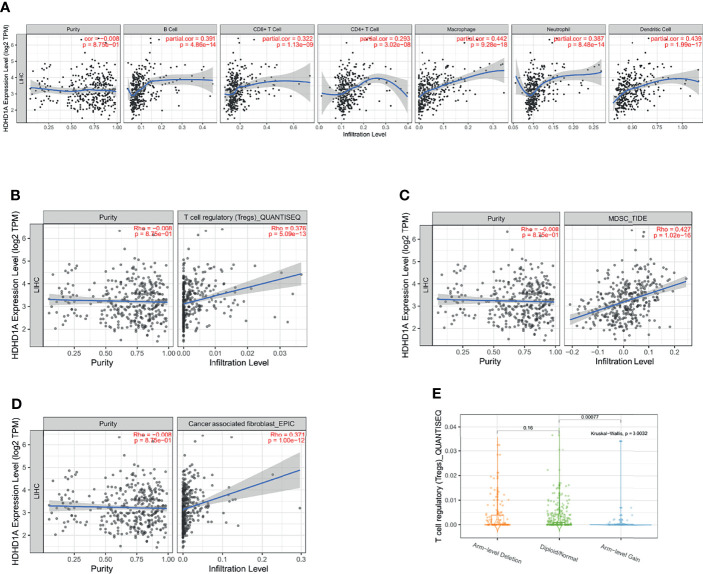
Exploring the relationship between the expression of PUDP and immune infiltration in HCC. **(A)** The correlation of PUDP and 6 common types of immune cells in HCC; **(B)** The correlation of PUDP and Tregs in HCC; **(C)** The correlation of PUDP and MDSC in HCC; **(D)** The correlation of PUDP and cancer associated fibroblast; **(E)** Tregs infiltration distribution between different sCNA status of the PUDP.

In recent years, with the advancement of research, it has become a consensus that the tumor immune microenvironment affects tumor progression. We analyzed the tumor purity of PUDP in HCC and its correlation with multiple immune cells. We found that PUDP showed a strong positive association with B cell, T cells regulatory (Tregs), CD8+ T cell, CD4+ T cell, Myeloid-derived suppressor cell (MDSC), macrophage, neutrophil, dendritic cell and cancer associated fibroblast (CAF) (**Figures 6A–D**). Surprisingly, there is a strong positive correlation between PUDP and Tregs, MDSC, and other immune cells that promote tumor progression. The sCNA information was derived from copy number segmentation profiles at the gene level. The alteration status for reference is high amplification. The results showed that the distribution of Tregs infiltration differed depending on the PUDP’s sCNA status (**Figure 6E**).

Moreover, we used the CellMiner database to investigate the relationship between PUDP and sensitivity to various anticancer chemotherapeutic drugs (**Supplementary Figure 3**). The results revealed a significant positive correlation between PUDP expression and sensitivity to tic10, nelarabine. It could shed new light on the clinical treatment of HCC.

### Correlation of PUDP Expression With Immunotherapy Response and Immune Checkpoints

In recent years, with the progression of immunotherapy, the prognosis of HCC patients has been improved, but some patients do not benefit from immunotherapy, so how to accurately distinguish which patients can benefit from immunotherapy is an urgent problem. Two distinct mechanisms of tumor immune escape: tumor-infiltrating cytotoxic T lymphocyte (CTL) dysfunction and CTL rejection by immunosuppressive factors were assessed by TIDE employing a set of gene expression markers. High TIDE scores are related with poor immune checkpoint blockade therapy (ICB) efficacy and short survival after ICB therapy. We used the TIDE algorithm to assess the responsiveness of the different PUDP expression groups to immunotherapy. The group with high PUDP expression had higher scores, suggesting that high PUDP expression patients with HCC may be less responsive to immunotherapy and not benefit from immunotherapy (**Supplementary Figure 4**).

In addition, we analyzed the molecular subtypes and immune subtypes of PUDP in HCC, and the results suggested that in HCC, the molecular subtypes of PUDP were mainly concentrated in iCluster:3, and the immune subtypes were mainly C1, C2, C3, C4 (**Supplementary Figure 5**).

**Figure 7:**
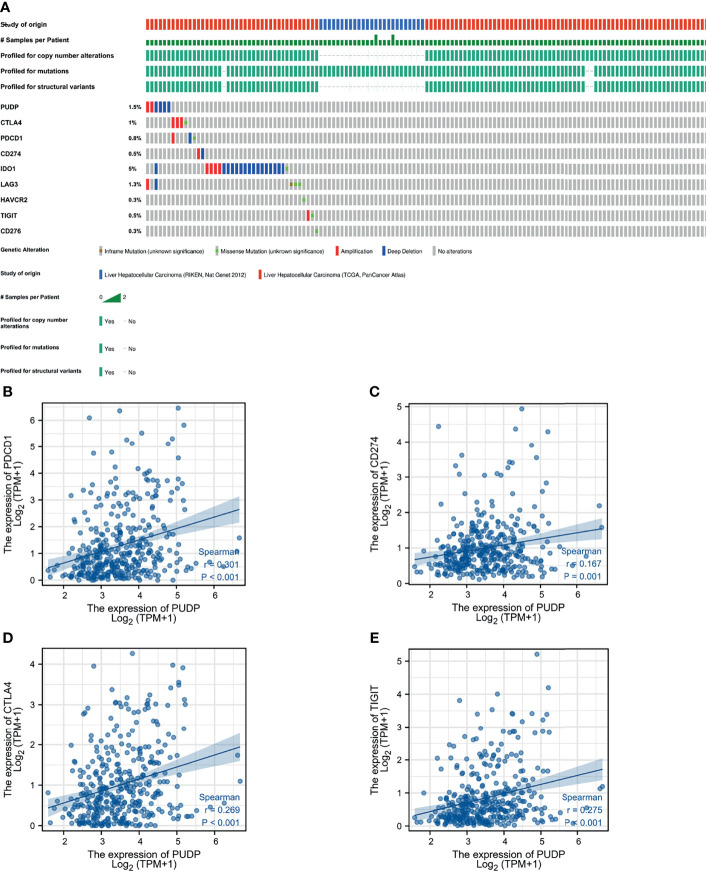
The relationship between PUDP and immune checkpoint genes. **(A)** Mutations in PUDP and common immune checkpoint genes in HCC. **(B–E)** Correlation analysis of PUDP with PD-1, PD-L1, CTLA-4, TIGIT respectively.

We also explored the correlation between PUDP and immune checkpoints mutation in HCC. The general landscape of PUDP and immune checkpoint alteration in HCC was compactly visualized, including inframe mutation, missense mutation, amplification, and deep deletion (**Figure 7A**). The detailed relationship between PUDP and common immune checkpoints in HCC was individually presented as indicated in **Figures 7B–E**.

### Analysis of PUDP Expression With Clinical Correlation and Methylation Modifications

**Figure 8:**
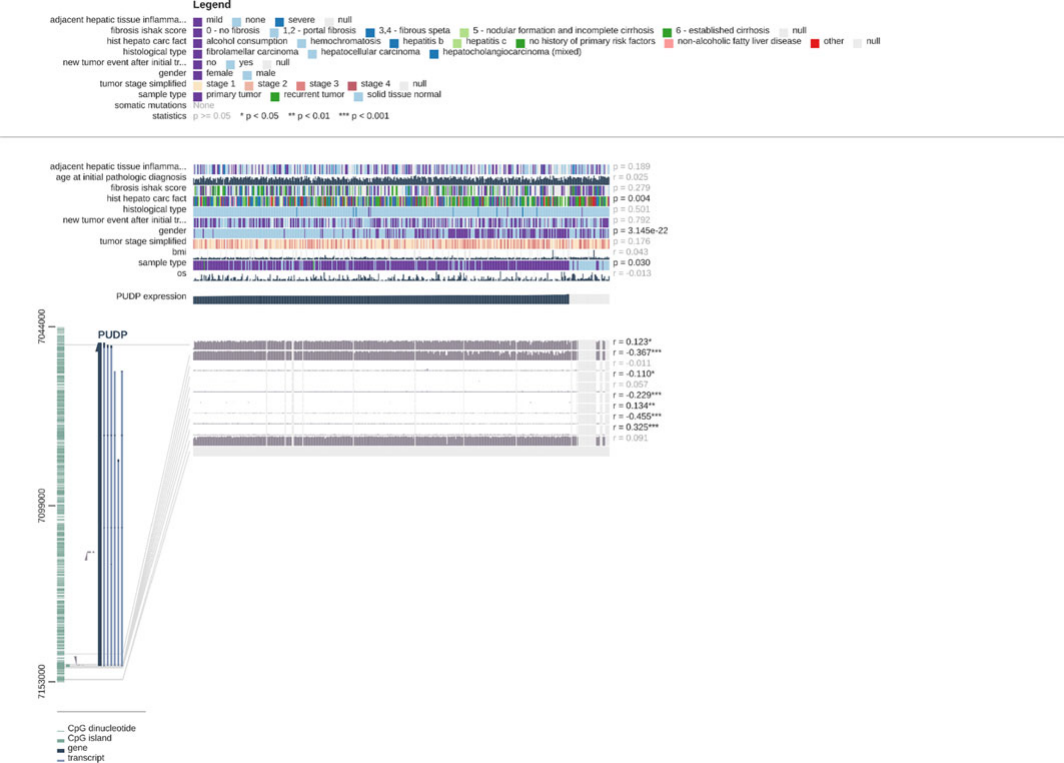
Correlations between PUDP expression and methylation modifications and the correlation of PUDP with clinical indicators.

We explored the clinical relevance of PUDP expression in HCC and found that the degree of PUDP expression was clinically significantly correlated with patient gender, and its prevalence was seen in primary HCC compared to the recurrent tumor and solid tissue normal. Meanwhile, we found that the methylation level of three loci on the X chromosome was positively correlated with PUDP expression, with probe ID: cg10858432, cg17878951, and cg26547788, respectively. And there were four loci on the X chromosome whose methylation levels were significantly and negatively correlated with the expression levels of PUDP, whose probe IDs were cg20214316, cg11024551, cg03043405, cg19788004 (**Figure 8**). Subsequently, we incorporated common clinical indicators and performed a logistic regression analysis with PUDP as the independent variable, PUDP low expression as the reference, and the detailed results are shown in **Table 2**. We also did subgroup K-M analysis in an attempt to find the detailed correlations between PUDP expression and clinical indicators. The results demonstrated that the association between high PUDP expression and prognosis was more pronounced in male than in female, while high PUDP expression indicates the poor prognosis in the early stage of HCC, and in addition, the association between elevated PUDP and prognosis was more statistically significant in patients without vascular invasion (**Supplementary Figure 6**).

**Table 2:** The relationship between PUDP and clinical indicators.

Characteristics	Total (N)	Odds Ratio (OR)	P value
Age (>60 vs. <=60)	373	0.731 (0.486-1.099)	0.132
Gender (Male vs. Female)	374	0.137 (0.080-0.226)	<0.001
T stage (T3&T4 vs. T1&T2)	371	1.855 (1.154-3.015)	0.011
N stage (N1 vs. N0)	258	2.817 (0.355-57.360)	0.373
M stage (M1 vs. M0)	272	3.000 (0.379-61.068)	0.344
Pathologic stage (Stage III&Stage IV vs. Stage I&Stage II)	350	2.144 (1.316-3.539)	0.002
AFP(ng/ml) (>400 vs. <=400)	280	4.811 (2.597-9.370)	<0.001
Tumor status (With tumor vs. Tumor free)	355	1.485 (0.975-2.269)	0.066
Histologic grade (G3&G4 vs. G1&G2)	369	2.489 (1.615-3.870)	<0.001
Albumin(g/dl) (>=3.5 vs. <3.5)	300	1.009 (0.589-1.733)	0.974
Vascular invasion (Yes vs. No)	318	1.252 (0.788-1.992)	0.341

### Exploring Predicted Upstream MiRNAs of PUDP

**Figure 9:**
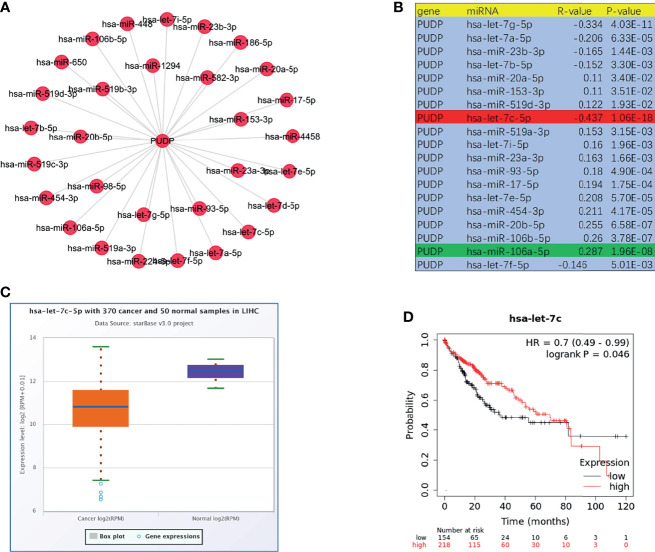
Prediction and analysis of upstream miRNAs of PUDP. **(A, B)** Potential upstream miRNAs that regulate the expression of PUDP; **(C)** Expression analysis of let-7c-5p in HCC; **(D)** Correlation analysis of let-7c-5p expression and prognosis of HCC patients.

There are widely accepted that miRNAs perform an important function in the regulation of gene expression and the development of malignancies. To determine whether PUDP was regulated by miRNAs, upstream miRNAs were predicted firstly and eventually we discovered 19 miRNAs that may potentially bind to PUDP. Using the cytoscape software, a miRNA-PUDP regulatory network was created to better visualization (**Figure 9A**). In HCC, PUDP was most strongly connected with let-7c-5p and miR-106a-5p, as shown in **Figure 9B**. Based on the mechanism of effect of miRNA in the regulation of target gene expression, let-7c-5p may be the promising candidate. Subsequently, the expression correlation analysis and survival analysis of let-7c-5p were performed. Let-7c-5p was significantly downregulated in HCC, as shown in **Figures 9C, D**, and its expression had significant correlation with patients’ prognosis. Finally, let-7c-5p expression and prognostic value in HCC were identified. All of these studies indicate let-7c-5p as the most promising PUDP regulatory miRNA in HCC.

### Clinical Staging and Cox Regression Analysis

**Figure 10:**
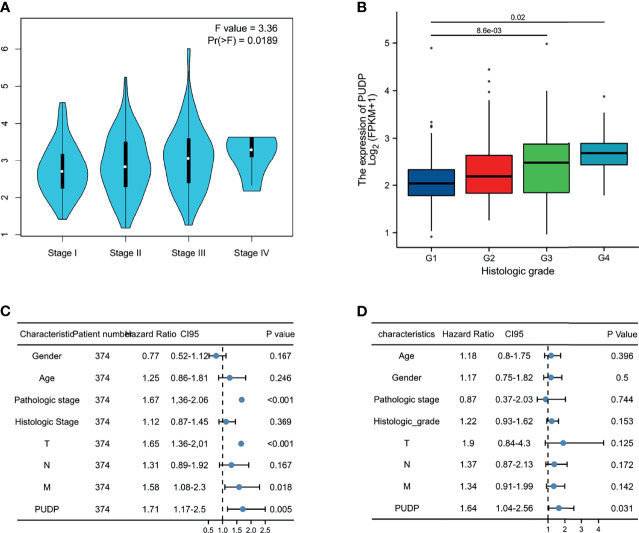
Expression of PUDP in different clinical stages and Cox regression analysis. **(A)** The expression of PUDP in different pathologic stages; **(B)** The expression of PUDP in different histologic grades; **(C, D)** Univariate and multivariate Cox regression analysis.

As the tumor progresses, whether there are differences in PUDP expression between patients with different clinical stages remains unknown, so we further explored the correlation between the expression of PUDP with different clinical stages, and the results showed that the expression of PUDP was significantly correlated with the pathologic stage and histologic grade (**Figures 10A, B**). It indicates that PUDP was significantly higher in patients with early-stage HCC and PUDP was differentially expressed in different staging HCC patients, suggesting that it may be a prognostic biomarker. To further explore the impact of the expression of the PUDP on HCC patient prognosis, Cox regression analyses were used and we found that the PUDP was an independent risk factor in HCC patients (*P* =0.031) (**Figures 10C, D**).

### Development of a Prognostic Model Based on PUDP Expression Profile

**Figure 11:**
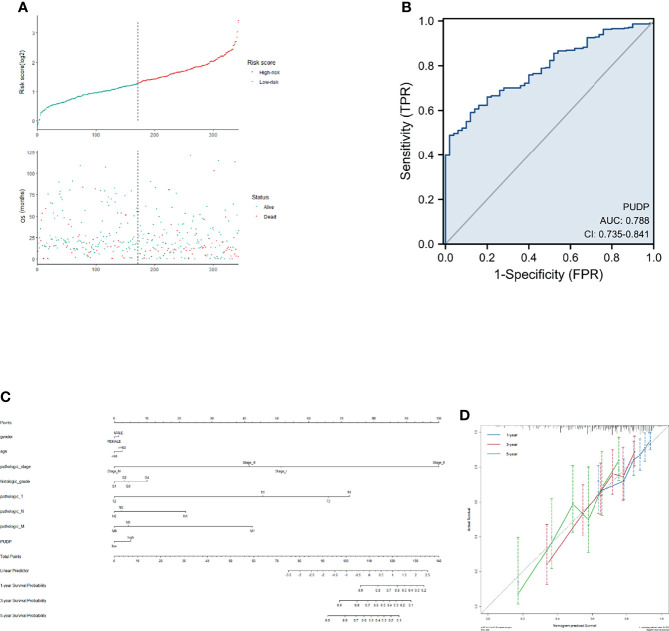
Construction of a prognostic model for HCC patients. **(A).** Risk factor correlation diagram; **(B)** ROC curve analysis; **(C)** A nomogram combining common clinical indicators with PUDP expression to predict 1-, 3-, and 5-prognosis survival; **(D)** Calibration curves of the nomogram, each curve was repeated 1000 times.

We incorporated the risk score and OS into the model to plot the risk factor diagram (**Figure 11A**). Then we constructed the nomogram to predict HCC patients’ OS (**Figure 11C**). C-index and the calibration curves were used as internal validation for the nomogram and C-index was performed to assess the discriminatory power of the nomogram (PUDP: 0.696 (0.668-0.724). Furthermore, the results of the calibration curves showed a good fit between the nomogram-predicted 1-, 3-, and 5-OS and the actual observed values (**Figure 11D**). We also used the ROC curve for evaluation, with an AUC value of 0.788 (**Figure 11B**). In addition, ICGC-JP dataset was employed as an external validation to assess the prognostic model’s accuracy and stability (**Supplementary Figure 6**).

## Discussion

HCC is one of the most prevalent malignancies in the world, and the vast majority of patients with HCC do not have a good prognosis. The results of a randomized study that included 600 untreated HCC patients showed that the overall median survival was 9 months, and the principal cause of death was tumor progression (30). Thus, it is urgent for us to find new and effective prognostic biomarkers.

In recent years, RNA modifications have gradually become the new frontier of cancer research. Common RNA modifications include N6-Methyladenosine, m6A methyltransferases, 5-Methylcytosine, Pseudouridine, etc (31). Pseudouridylation, in particular, is known to be the most abundant modification in total RNA from human cells, but the effect of Pseudouridylation is still poorly understood. Previous studies have shown that RNA pseudouridine modification could affect the prognosis of glioma patients by enriching tumour-infiltrating immune cells such as macrophages M0 and Tregs (32). Dyskerin pseudouridine synthase 1 (DKC1), a gene located on the X chromosome, is associated with the progression of multiple diseases (33–36). A previous study demonstrated that elevated levels of reactive oxygen species (ROS) in HCC regulate cytoplasmic protein-disulfide isomerase-associated 3 (PDIA3) levels, leading to HCC cell survival through upregulation of DKC1. Similar to DKC1, PUDP is also involved in the pseudouridine process and is located on the X chromosome. However, we are still unclear about the biological function of PUDP. In the present study, we systematically investigated the possible mechanisms and biological functions of PUDP in the development of HCC.

Pan-cancer expression analyses of PUDP were performed firstly and we found that PUDP was significantly highly expressed in most tumor tissues. It suggested that PUDP may be involved in the development of tumors. Investigations of the correlation between PUDP expression level and tumor patients’ prognosis were explored and the results demonstrated PUDP was significantly negatively correlated with OS in patients with HCC and PAAD. Especially in HCC patients, high PUDP expression was strongly negatively correlated with OS, DSS, and PFI of patients. For exploring the expression of PUDP in HCC patients, we included two GEO datasets (GSE39791, GSE25097) to perform expression analysis and tumor tissues with paired non-tumor tissues from 15 HCC patients from the tumor tissues bank of Xijing Hospital for qPCR to detect their RNA expression levels, and finally used the HPA database to identify the protein expression of PUDP in HCC patients. All of the results showed that the expression level of PUDP was significantly increased in HCC tissues compared with normal tissues.

To further investigate the role of PUDP in HCC, we divided the HCC patients into PUDP high expression group and PUDP low expression group, and identify the DEGs between the two groups. Co-expression genes are involved in the development of multiple cancers, for example, high expression of SMR3A suggests poor prognosis in patients with head and neck squamous cell carcinoma, and WNT7B promotes gastric cancer progression and metastasis (37, 38). We also found that co-expression genes are mainly involved in antigen binding, neuroactive ligand-receptor interaction, bile secretion pathways.

In recent years, with in-depth research, it has become a consensus that the tumor immune microenvironment is involved in tumor progression. In order to investigate the relationship between PUDP and tumor immune microenvironment, we analyzed the correlation between PUDP and common immune cells in HCC. First, we analyzed the scRNA sequencing data of HCC patients (GSE125449), which showed that PUDP was expressed on CD8Tex, Mono/macrophages, endothelial, fibroblasts, malignant, hepatic progenitor cells, especially on malignant cells with significantly high expression.

Then, we explored the correlation between the expression level of PUDP and various immune cells such as Tregs, CD8+ T cells, MDSCs, CD4+ T cells, macrophages, and CAFs et al. Interestingly, we found that the expression of PUDP was significantly positively correlated with the expression of Tregs, MDSCs, CAFs. Tregs, one subset of T lymphocytes that regulate the immune response according to suppressing the effector T lymphocytes (39, 40). Tregs have been shown to promote tumor development, for example, in non-alcoholic steatohepatitis (NASH) -associated HCC, Tregs interact with neutrophil extracellular traps (NETs) to inhibit immune surveillance to exert oncogenic effects in the early stage of NASH, while in HBV-associated HCC, specifically blocking the infiltration of Tregs could enhance the anti-tumor immunity and Tregs expression correlated with sorafenib resistance and HBV load titers (41, 42). In cancer, the differentiation of myeloid cells is often altered, producing a cohort of immature myeloid cells called MDSCs that have strong immunosuppressive activity and impaired function as antigen-presenting cells (APCs) (43). MDSCs impede dendritic cell function, inhibit T-cell tumor invasion, and reduce the efficacy of current immune checkpoint treatments. Drug-resistant HCC cells have been found to perform the cancer stem cell (CSC) characteristics, which can boost MDSC proliferation and immunosuppressive activity by preferentially secreting IL-6 (44). In the HCC tumor microenvironment, activated hepatic stellate cells (HSCs) are the major source of CAFs and the crosstalk between activated HSC/CAF and tumor cells is associated with tumor cell proliferation, migration, metastasis, and chemoresistance (45). Given that more than 80% of HCC tumors develop in the setting of cirrhosis, which in turn is associated with a large number of activated fibroblasts due to the presence of chronic inflammation, CAFs are known to be critical to the development and progression of HCC and have a significant impact on the clinical outcome of patients (46). According to our findings, PUDP is strongly and positively correlated with a range of tumor-promoting immune cells such as Tregs, MDSCs, and CAFs, suggesting that PUDP may influence the progression and prognosis of patients with HCC by interacting with immunosuppressive cells in the tumor microenvironment. For the mutation of PUDP in HCC patients, we have studied, and the results showed that the mutation form of PUDP in HCC patients existed mainly in amplification, deep deletion.

Furthermore, immunotherapy success is dependent not only on sufficient immune cell infiltration in the TME, but also on enough expression of immune checkpoints. Therefore, the relationship between PUDP and immune checkpoints was analyzed and the results showed that PUDP was significantly and positively correlated with the expression of TIGIT, PD-1, PD-L1, CTLA-4, and other immune checkpoint genes, and the highest correlation was with the expression of the PD-1 gene (*r=0.301, p <0.001*). Despite the remarkable clinical success of immune checkpoint blockade therapy, most patients do not benefit from immunotherapy (47–49). It is, therefore, crucial to accurately distinguish which patients can benefit from immunotherapy. Using the TIDE algorithm, we found that patients with high PUDP expression were less likely to benefit from ICB. For the first time, we analyzed the correlation between PUDP and therapeutic sensitivity to anticancer drugs. The expression of the PUDP gene was significantly positive with tic10, Nelarabine and negative with AZD-1480, Dovitinib, ITRI-260. Previous studies demonstrated that Dovitinib inhibits the metastasis and invasion of HCC through the antiangiogenic mechanism, and overcomes sorafenib resistance (50, 51).

It has been well documented that miRNAs were involved in the regulation of gene expression (52–54). PITA, RNA22, miRmap, microT, miRanda, PicTar, and TargetScan were used to predict the upstream regulatory miRNAs of PUDP. 19 miRNAs were finally obtained. In HCC, the majority of miRNAs have been found to function as tumor suppressor, for example, upregulated let-7a-5p could prevent HCC progression (55). In the end, the most potential upstream tumor suppressor miRNA for PUDP was identified as let-7c-5p after correlation analysis, expression analysis, and survival analysis. Previous studies demonstrated that sponging let-7c-5p could promote proliferation and migration of HCC (56, 57).

In addition, the relationship between the expression of PUDP and clinical indicators in HCC patients was analyzed using the MEXPRSS database and TCGA HCC cohort. The results revealed that high PUDP expression was common in primary HCC, and there were gender differences. The results of subgroup K-M analysis showed that high PUDP expression in male compared to female was more significant in the prognosis of patients with HCC. We also performed a subgroup K-M analysis for clinical staging and clinical-grade. The results demonstrated high expression of PUDP in the early stage of HCC could indicate a poor prognosis. By incorporating PUDP expression with common clinical indicators, Cox regression analyses were performed, identifying high PUDP expression was an independent risk factor for HCC patients.

This study still has some areas for improvement. First, we lack a prospective clinical cohort study to explore the detailed correlation between PUDP expression and the prognosis and clinical stage of patients with HCC. Second, although based on the previous studies, we can speculate that PUDP expression in serology is consistent with changes in expression in tissues, however, we lack serological data to validate. Finally, the prevalence of COVID-19 has made access to the laboratory very difficult, thus lacking validation of the detailed mechanisms of PUDP *in vitro* and *vivo*.

## Conclusion

In summary, our findings suggested that PUDP was highly expressed in most tumors and high expression of PUDP indicated the poor prognosis and low response to immunotherapy in HCC. The most potential upstream miRNA of PUDP was identified as let-7c-5p. Mechanistically, we found that PUDP has a strong positive correlation with tumor-promoting immune cells such as Tregs, MDSCs, CAFs. Moreover, there are significantly positive correlations between the PUDP expression and immune checkpoint. These findings enhanced our understanding of PUDP and suggested that PUDP may exert oncogenic effects by the enrichment of tumor immune cell infiltration and expression of immune checkpoints. Finally, the prognostic model was constructed by the data from TCGA and validated by the data from ICGC.

## Data Availability Statement

The original contributions presented in the study are included in the article/**Supplementary Material**. Further inquiries can be directed to the corresponding authors.

## Ethics Statement

The studies involving human participants were reviewed and approved by Xijing Hospital’s Ethics Committee. The patients/participants provided their written informed consent to participate in this study.

## Author Contributions

Conceived and designed the experiments, YH, JW, and GG. Collected the data, WZ, JY, and YNH. Analyzed the data, JY, YNH, DD, and WZ. Drafted the manuscript, JY, YNH, WZ, and DD. All authors contributed to the article and approved the submitted version.

## Funding

The study was funded by National Key Research and Development Program of China (2020YFA0710803, 2017YFA0105704), National Natural Science Foundation of China (81900502, 81820108005, 81870421, 81873562 and 81870398), Key Research and Development Projects of Shaanxi Province, 2021ZDLSF02-07.

## Conflict of Interest

The authors declare that the research was conducted in the absence of any commercial or financial relationships that could be construed as a potential conflict of interest.

## Publisher’s Note

All claims expressed in this article are solely those of the authors and do not necessarily represent those of their affiliated organizations, or those of the publisher, the editors and the reviewers. Any product that may be evaluated in this article, or claim that may be made by its manufacturer, is not guaranteed or endorsed by the publisher.
