# Supplementary material for: Bioinformatics Analysis Combined With Experiments Predicts PUDP as a Potential Prognostic Biomarker for Hepatocellular Carcinoma Through Its Interaction With Tumor Microenvironment
Source: Front Oncol. 2022 Mar 8;12:830174. doi: 10.3389/fonc.2022.830174 (PMC8957838; doi:10.3389/fonc.2022.830174)
Supplement: Supplementary file 1 [file DataSheet_1.docx]

**Supplementary Figure 1. The gene set enrichment analysis (GSEA) of co-expression genes.**


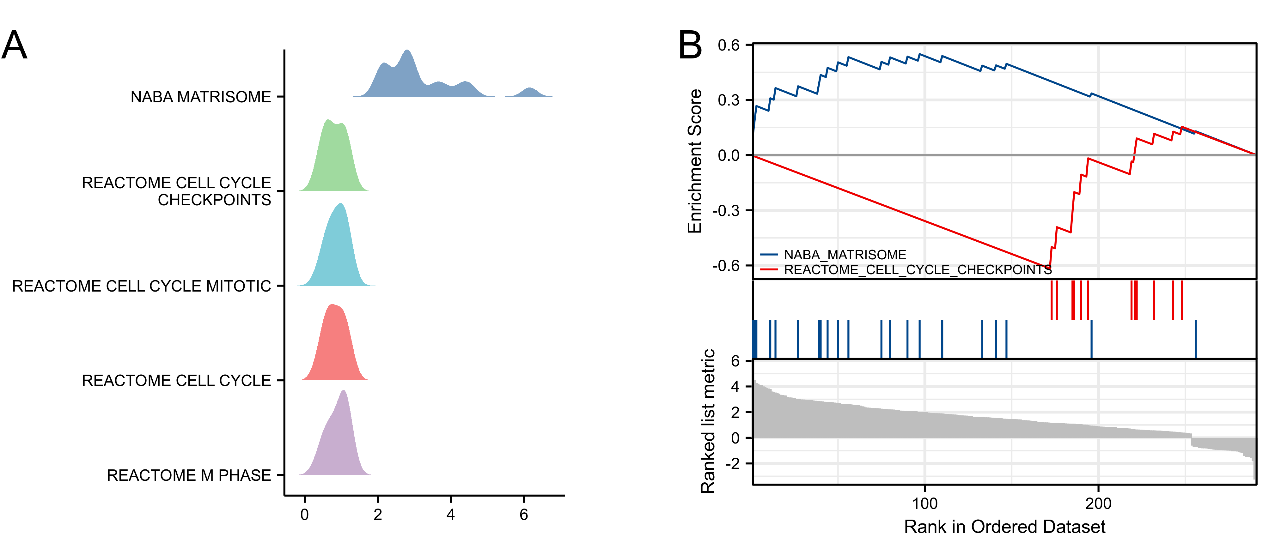


**Supplementary Figure 2. The analysis of the expression of PUDP on the cell in the tumor microenvironment**

**
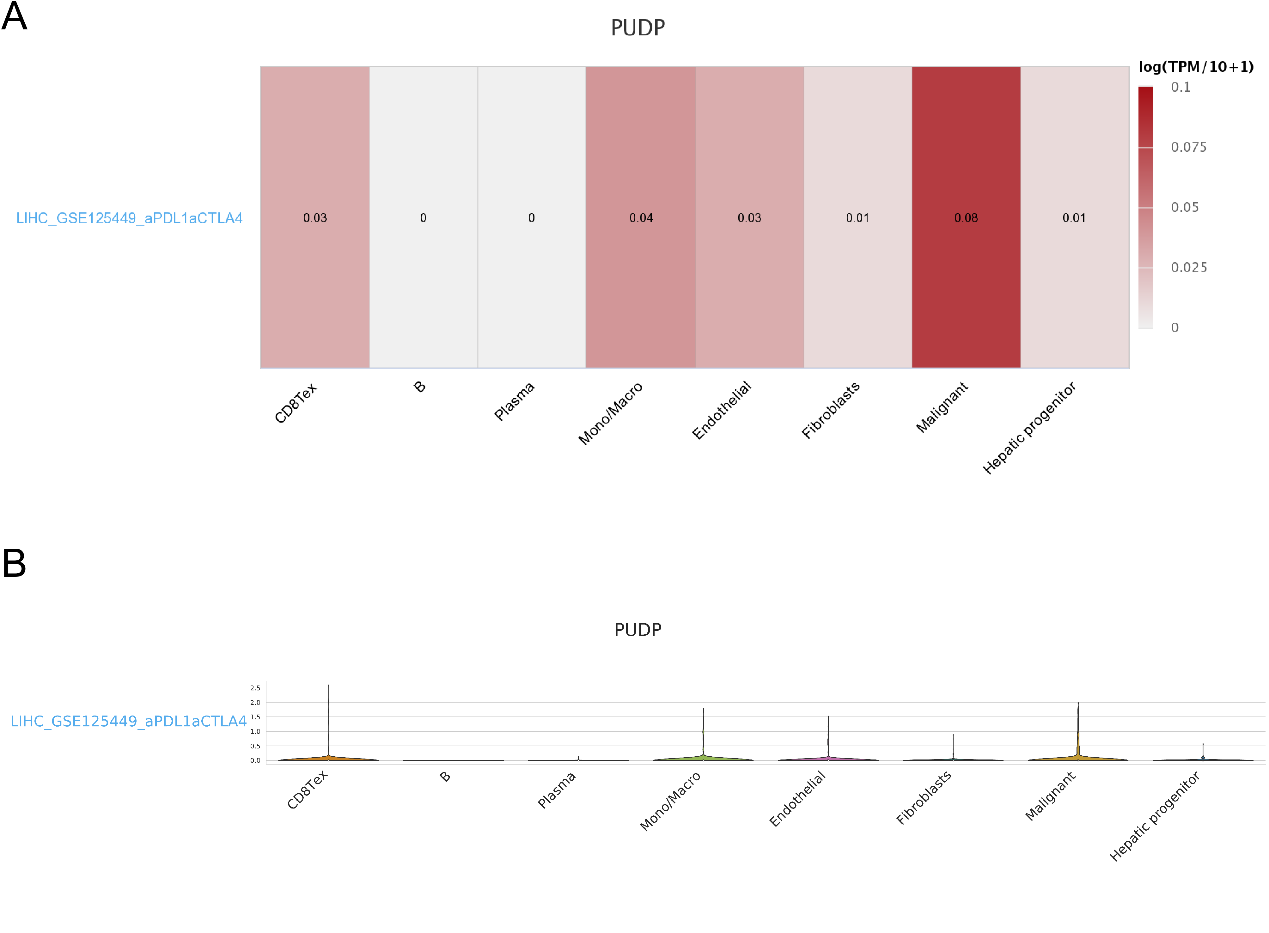
**

**Supplementary Figure 3. The analysis of sensitivity of PUDP to anticancer drugs based on CellMiner database.**

**
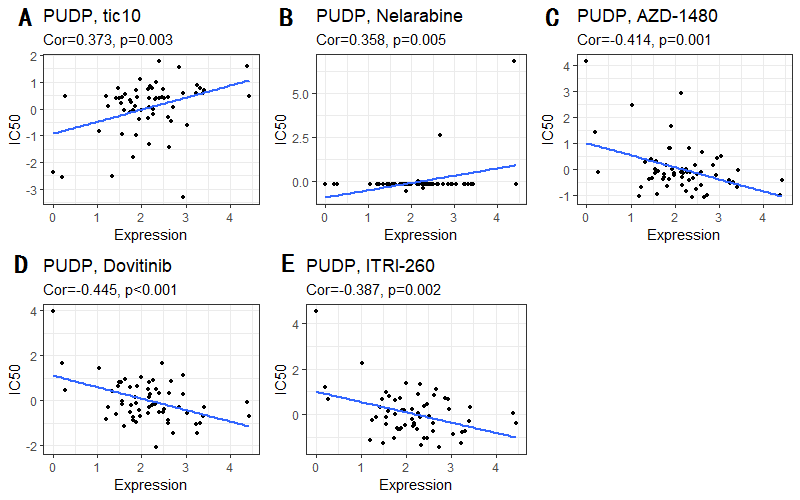
**

**Supplementary Figure 4. The analysis of immunotherapeutic response between high expression of PUDP and low expression of PUDP in HCC patients.**

**
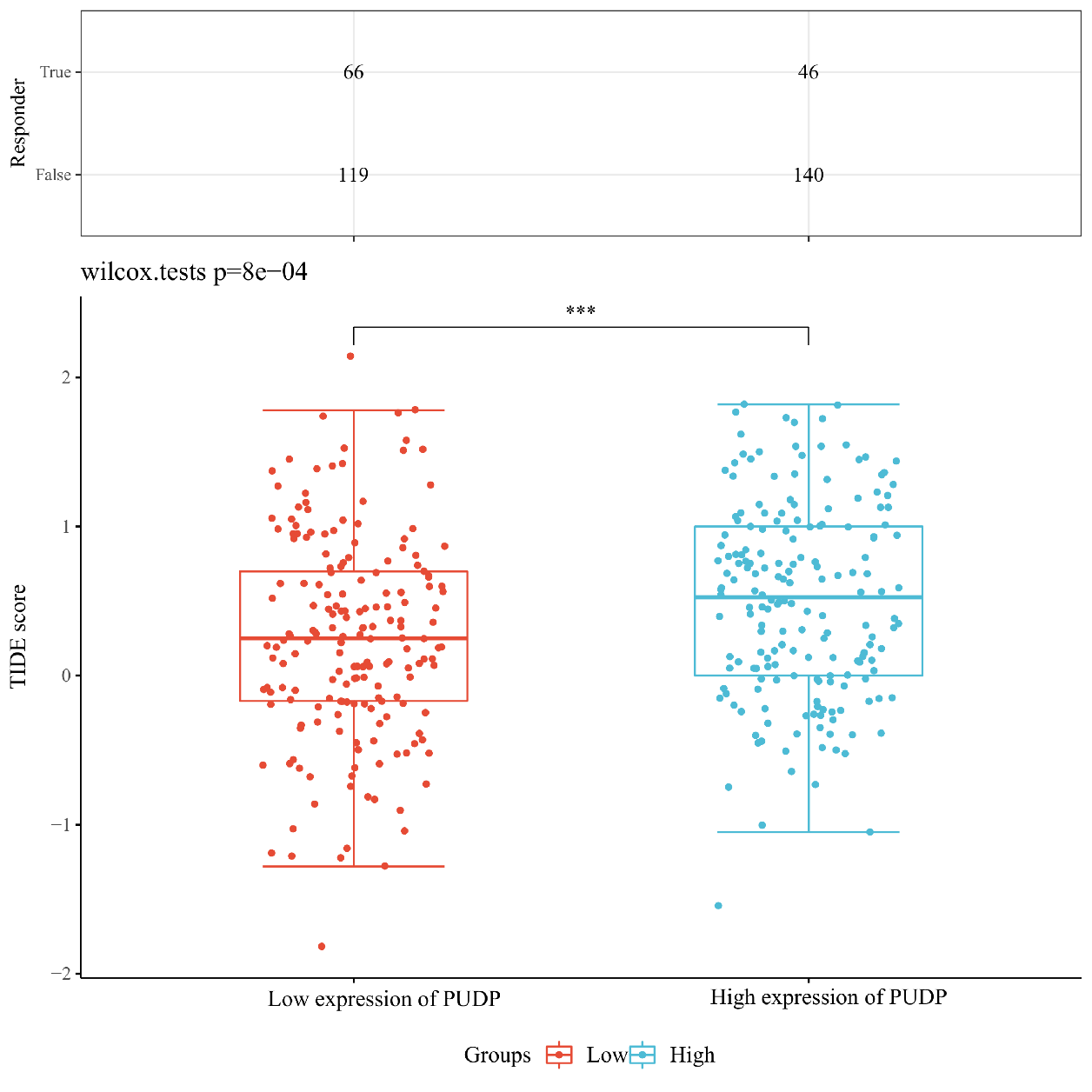
**

**Supplementary Figure 5. The analysis of molecular subtypes and immune subtypes of PUDP in HCC.** (A) Immune subtypes of PUDP ;(B) Molecular subtypes of PUDP.

**
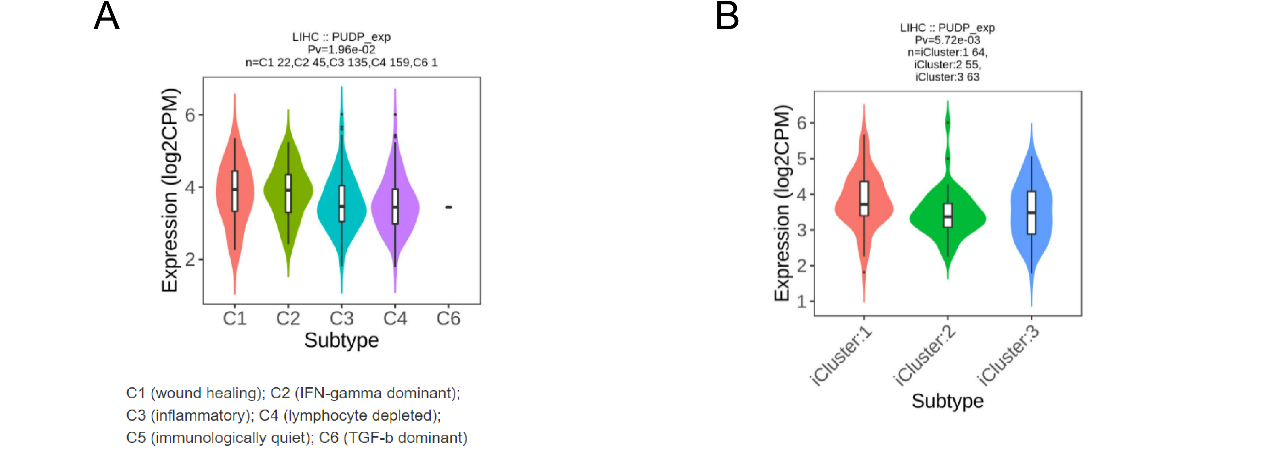
**

**Supplementary Figure 6. Subgroup survival analysis of prognosis and PUDP expression in patients with HCC.**

**
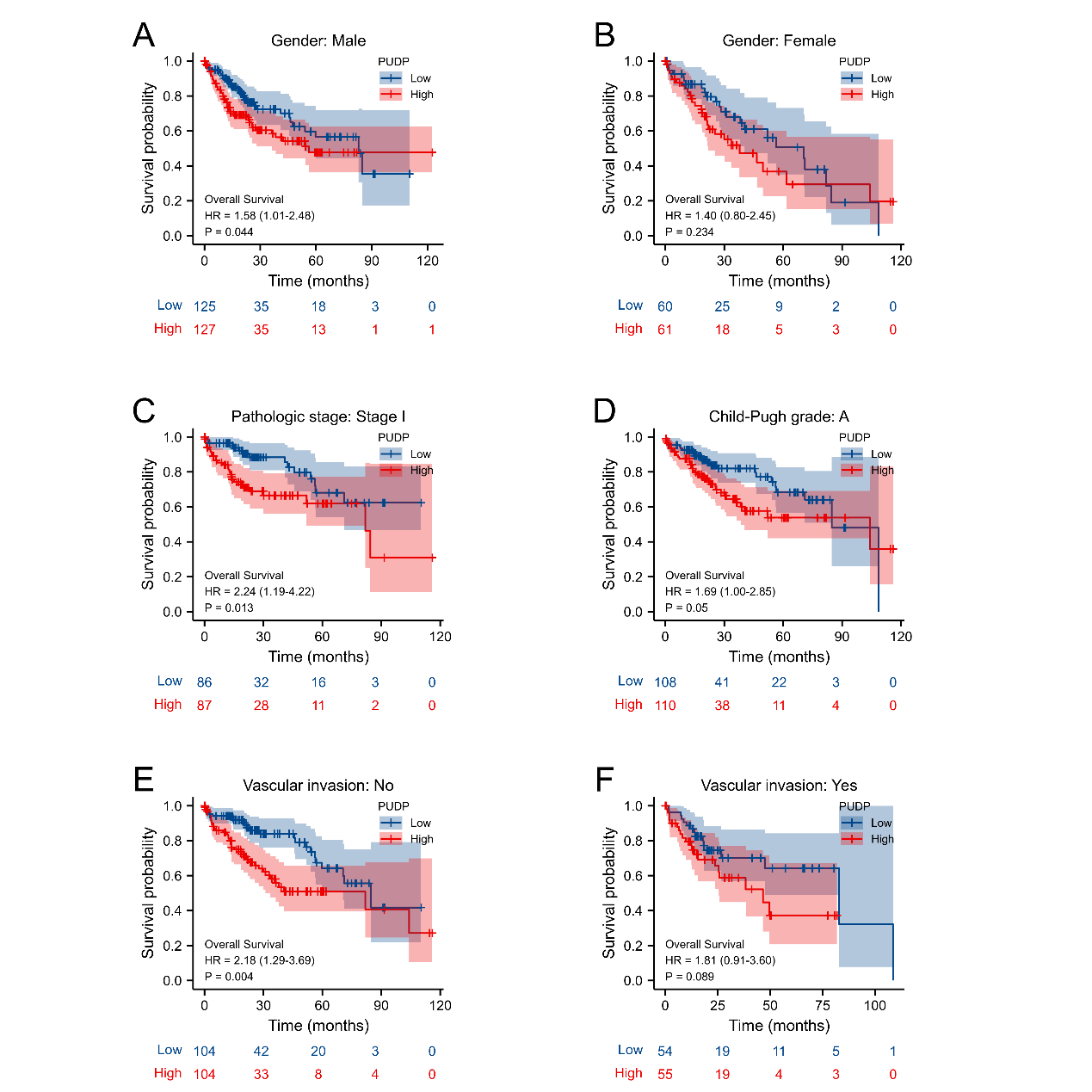
**

**Supplementary Figure 7. External Validation of the prognostic models using the data from ICGC.**

**
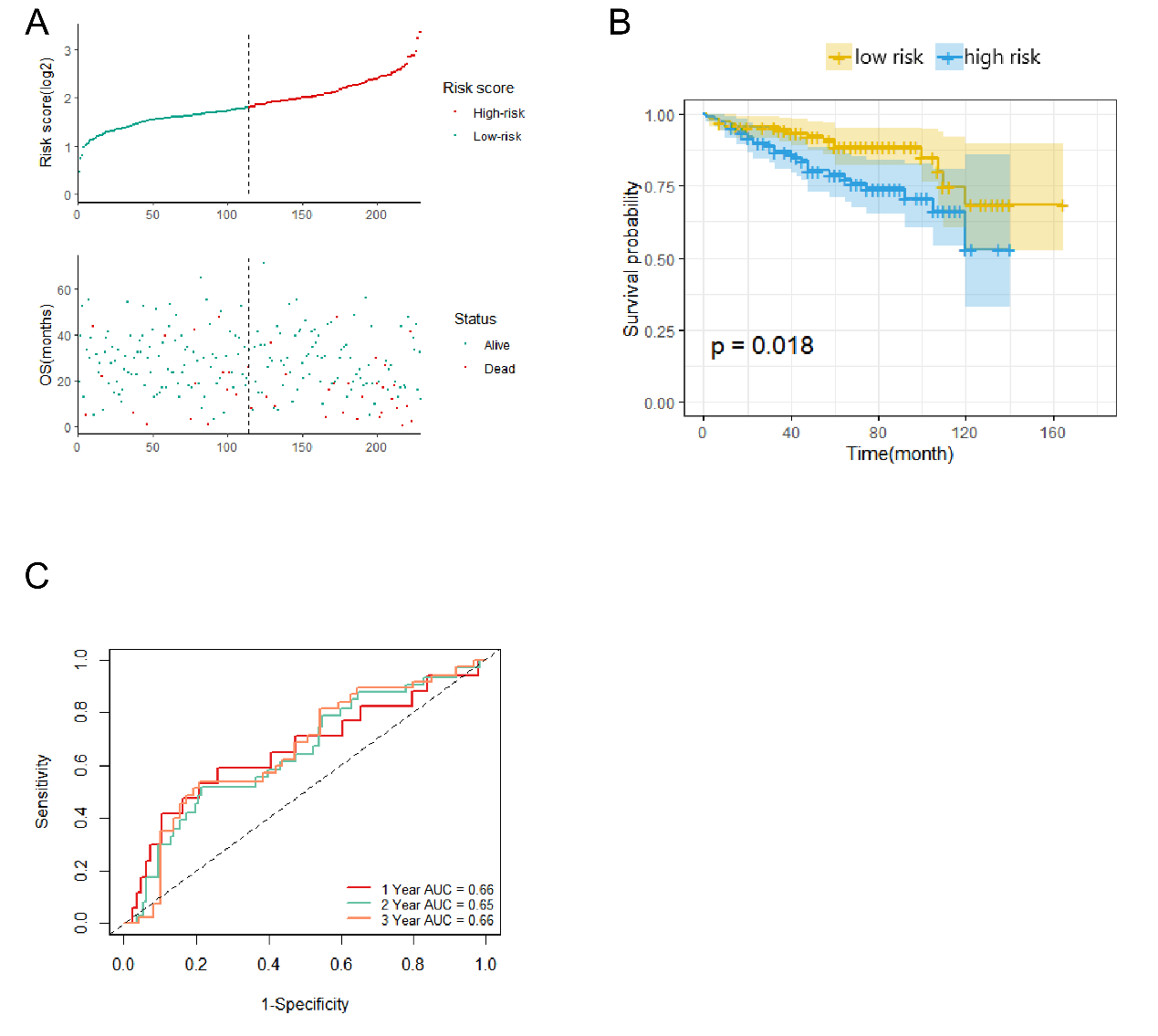
**
